# “Asymptomatic” Malaria: A Chronic and Debilitating Infection That Should Be Treated

**DOI:** 10.1371/journal.pmed.1001942

**Published:** 2016-01-19

**Authors:** Ingrid Chen, Siân E. Clarke, Roly Gosling, Busiku Hamainza, Gerry Killeen, Alan Magill, Wendy O’Meara, Ric N. Price, Eleanor M. Riley

**Affiliations:** 1 Global Health Sciences, Malaria Elimination Initiative, University of California, San Francisco, San Francisco, California, United States of America; 2 Department of Disease Control, Faculty of Infectious and Tropical Diseases, London School of Hygiene & Tropical Medicine, London, United Kingdom; 3 Ministry of Health, National Malaria Control Centre, Lusaka, Zambia; 4 Liverpool School of Tropical Medicine, Vector Biology Department, Liverpool, United Kingdom; 5 Ifakara Health Institute, Ifakara, Morogoro, United Republic of Tanzania; 6 Bill & Melinda Gates Foundation, Seattle, Washington, United States of America; 7 Duke Global Health Institute, Duke University, Durham, North Carolina, United States of America; 8 Global and Tropical Health Division, Menzies School of Health Research and Charles Darwin University, Darwin, Australia; 9 Centre for Tropical Medicine and Global Health, Nuffield Department of Clinical Medicine, University of Oxford, Oxford, United Kingdom; 10 Department of Immunology and Infection, Faculty of Infectious and Tropical Diseases, London School of Hygiene & Tropical Medicine, London, United Kingdom

## Abstract

Roland Gosling and colleagues argue that "asymptomatic" malaria infections have significant health and societal consequences, and propose that they should be renamed "chronic" malaria infections.

**Summary Points**

- Are afebrile malaria infections truly asymptomatic, benign, or even beneficial to the individual? The evidence suggests the contrary.
- So-called “asymptomatic” malaria infections are associated with recurrent episodes of symptomatic parasitemia, chronic anemia, maternal and neonatal mortality, co-infection with invasive bacterial disease, cognitive impairment, and ongoing transmission of the parasite.
- “Asymptomatic” malaria infections have significant health and societal consequences, and we propose that they should be renamed “chronic” malaria infections.
- Targeting chronic malaria infections poses major scientific, operational, and ethical challenges.
- We call for the malaria community to work with malaria control and elimination programs to target all malaria infections, irrespective of their density or presentation. The operational challenges to detect and treat chronic infections are significant, but accomplishing this is likely to result in substantial gains to both the individual and society.

## Background

Since ancient times, the concept of malaria has been synonymous with its most obvious symptoms: a characteristic recurrent cycle of fevers and chills. Obvious symptoms and severe disease mostly occur in partially immune or non-immune individuals, especially children in high-transmission settings and visitors to endemic areas. So-called “asymptomatic” malaria infections have been recognized for many years, and result from partial immunity (sometimes referred to as “premunition”), which controls but does not completely eliminate the infection. Thus, persistent or repeated “asymptomatic” infection is sometimes viewed as beneficial to the individual, as it helps to maintain this state of premunition, thereby reducing the risk of severe disease [1]. In this paper, we present evidence to the contrary: persistent infections with malaria parasites are frequently detrimental to the individual, with serious health, developmental, and productivity consequences. We propose they should be described more accurately as “chronic” infections requiring curative treatment.

Although there is no standard definition for “asymptomatic” malaria infections, it is generally accepted to be malarial parasitemia of any density, in the absence of fever or other acute symptoms, in individuals who have not received recent antimalarial treatment [2]. At any given time, the vast majority of individuals with detectable malaria parasitemia can be categorized as asymptomatic based on this definition, regardless of the level of malaria transmission [3]. This definition includes early detection of rising parasitemia that has yet to reach the pyrogenic threshold (i.e., the density of parasitized erythrocytes that is sufficient to trigger innate immune responses and fever) [4], infections that are intermittently symptomatic but not severe enough to cause the person to seek health care [5], and long-standing infections imperfectly controlled by the immune response. Current guidelines aim to improve the early detection of new symptomatic and subacute cases, for example, through the use of village health workers [6], but this strategy does not reach individuals carrying low-density, chronic infections. Chronic infections tend to be of substantially lower density than acutely symptomatic infections; indeed, they can be “submicroscopic” (i.e., not detected on a blood film or by rapid diagnostic test [RDT] but detectable by more sensitive tests such as PCR) and can persist for several months or even years [4].

An important question is whether “asymptomatic” malaria infections are truly benign and, if not, whether the advantages of premunition are outweighed by the harm that arises from persistent blood-stage parasites. If so, are there particular high-risk groups, in addition to young children and pregnant women, which need to be identified? From a public health perspective, the risks and benefits need to be examined with regard not just to the individual’s well-being but also within the wider context of sustainable malaria control strategies that may lead to elimination and eradication.

In this policy forum article, we propose that, notwithstanding the policy and programmatic challenges that will arise, treating all malaria infections, irrespective of their clinical presentation, will confer direct clinical benefits to the individual and may contribute to improved malaria control at the population level by reducing onward transmission. This paper is a collection of four strands of evidence supporting our argument: that malaria infections of any density are important to find and to treat.

## Evidence

### Malaria Control Interventions Reduce All-Cause Morbidity and Mortality

It has long been suspected that infection with malaria parasites at any density of infection may exacerbate other diseases, and, thus, reducing the prevalence of malaria will reduce mortality and morbidity ascribed to other causes. At an individual level, the relationship between parasitemia, morbidity, and mortality is complicated by the fact that while malaria infection can be the primary causal agent, it can also be a contributing co-morbidity, or it can be coincidental and unrelated to the main cause of illness. At a population level, changes in all-cause, rather than malaria-specific, morbidity and mortality under effective control strategies offer insight into the contribution of malaria as an indirect cause of hospitalization, illness, and death.

Observational evidence from Kilifi, Kenya, showed that there was an excess reduction in all-cause hospital admissions as malaria transmission in the area declined [7]. The reduction in all-cause hospital admissions was greater than that expected from the decline in malaria admissions alone based on estimation of the malaria-attributable fraction (the proportion of malaria episodes in which parasites are responsible for the fever, estimated by comparing parasite density in asymptomatic infections to parasite density in symptomatic cases). The decline in malaria episodes was accompanied by a concurrent decline in invasive bacterial disease [8], supporting the notion that malaria infection predisposes individuals to bacteremia (see below).

Among malaria intervention trials that report all-cause mortality as an outcome, the reduction in overall mortality consistently exceeds that of malaria-specific mortality [9–12]. Two seminal insecticide treated bed-net (ITN) trials, one in The Gambia and one in Ghana, showed significant reductions in all-cause mortality of 20%–25% amongst children protected by ITNs but no significant differences in malaria-specific mortality [9,10]. Similarly, a chemoprophylaxis study in The Gambia demonstrated marked reductions in all-cause mortality after just one year of chemoprotection [11]. The reduction in all-cause mortality was more than twice the reduction in malaria-specific mortality after one year and three to four years of protection [11,12]. These findings clearly implicate undiagnosed “asymptomatic” malaria infections as a component of some multifactorial causes of death.

Impact on morbidity is more complex. The reduction in all illness episodes in infants receiving malaria chemoprophylaxis from birth was twice the reduction in malaria-specific outpatient events [13]. A chemoprophylaxis trial in children under five years of age also demonstrated a reduction in all-cause morbidity, including lower rates of gastrointestinal and respiratory illness, although the effect was only apparent after three to four years of chemoprotection and was less than the change in malaria-specific morbidity [12,14]. Conversely, a chemoprophylaxis study in schoolchildren in a region of moderate to high malaria endemicity demonstrated no reduction in all-cause hospitalization after accounting for reductions in malaria episodes [15]. Taken together, the impact of reducing malaria infection on other causes of morbidity may be most apparent in non-immune populations (i.e., in infants or in children in low-transmission areas) and, perhaps, in those chemoprotected for several years.

### Chronic Malaria Infections, Anemia, and Pregnancy

While the impact of malaria infections on all-cause mortality is evident, the underlying reasons for this relationship are far from clear. The direct clinical manifestations of peripheral parasitemia are driven by the magnitude of the infecting biomass, the species and the chronicity of infection. Individuals with a higher peripheral parasitemia are at greater risk of being febrile since the pyrogenic threshold is more likely to be exceeded. This threshold varies according to levels of acquired immunity, being much higher in partially immune individuals than in those without immunity, and lower in *Plasmodium vivax* infections compared to *Plasmodium falciparum* infections [16]. Since treatment-seeking only occurs after the onset of symptoms, semi-immune individuals tend to present later, if at all, compared to non-immune individuals, resulting in prolonged carriage of low-density parasitemia. Low-level “asymptomatic” malaria can result in chronic, low-grade hemolysis as well as intermittent, higher density symptomatic recurrences [17]. Each recurrent episode of symptomatic malaria causes a further bout of hemolysis, with 8%–14% loss of red blood cell mass [18]. The greater the number of symptomatic recurrences and the higher the parasitemia, the greater the degree of red cell destruction and the greater the fall in hemoglobin; the longer this goes on, the greater the impairment of red cell production [19], the more extensive the destruction of nonparasitized red cells [20,21], and the greater the cumulative burden of anemia [22].

Recurrent parasitemia is also associated with splenomegaly arising from filtration, retention, and phagocytosis of parasitized erythrocytes; erythrocyte debris and damaged or dysfunctional uninfected red cells; and from the associated inflammatory response [23]. In extreme cases, hyper-reactive malarial splenomegaly (HMS) can arise from chronic antigenic stimulation secondary to malaria parasitemia, resulting in hemolytic anemia, splenic rupture, and increased susceptibility to other acute infections [24].

In areas where “asymptomatic” malaria is highly prevalent, defining malaria-attributable anemia can be challenging [25]. Helminth infections and hemoglobinopathies are common and can exacerbate anemia whilst simultaneously providing a degree of protection from symptomatic malaria [26,27]. In resource-poor settings, infants and malnourished individuals are susceptible to helminth infection and iron deficiency; any additional hematological insult, including malarial hemolysis, can tip the individual into significant clinical anemia. A subsequent rapid fall in hemoglobin concentration during an acute episode of symptomatic malaria can lead to hemodynamic compromise (decompensation). As the degree of anemia increases, the risk of decompensation, mortality attributable to parasitemia, co-morbidities, and bacterial co-infection rises exponentially [17].

“Asymptomatic” malaria and placental parasitemia also have major consequences for mothers and their newborns. The vast majority of women with placental malaria have no obvious clinical signs of malaria infection during their pregnancy, and circulating chronic infections are a major source of placental infection [28,29]. Placental malaria infection is associated with placental inflammation, fibrosis, and functional insufficiency, leading directly to miscarriage, preterm delivery, low birth weight, and peripartum hemorrhage and, thus, increased maternal and neonatal mortality [30,31].

### Malaria Infection Increases the Risk of Systemic Bacterial Infections

Despite abundant evidence that “asymptomatic” malaria infection contributes to all-cause mortality and morbidity, the underlying pathologies associated with this remain poorly described. There are numerous reports of dysregulated immune responses in individuals with malaria—at any density of infection [32–34]—but these have rarely been linked mechanistically to specific disease outcomes. However, malaria parasitemia does clearly lead to one specific immunological defect that has important clinical consequences: an increased risk of invasive bacterial disease. Associations between bacteremia (especially non-typhoid salmonellae, NTS), concurrent malaria infection, and anemia have been noted since the 1920s, but the first detailed analysis of a large case series was published from The Gambia in 1987. Seventy percent of patients with NTS bacteremia were also parasitemic and anemic, typically with “asymptomatic,” very low parasite densities [35]. This association between NTS bacteremia, recent or current low density *P*. *falciparum* parasitemia, and mild to moderate anemia has since been demonstrated numerous times [36], and epidemiological evidence strongly supports a causal association.

Invasive NTS infections are much more common in areas of intense malaria infection than in low malaria transmission areas [37], temporal declines in malaria transmission intensity have been accompanied by similar declines in the incidence of invasive NTS infections [38,39], and carriage of the sickle cell trait reduces the risk of bacteremia by 64% in Kenyan children [8]. A systematic review of community-acquired bacteremia in Africa revealed that there are approximately 1 million cases of NTS bacteremia in sub-Saharan Africa every year, with an in-hospital case fatality rate of almost 20% [40]. Scott et al. [8] estimate that over 60% of these cases are attributable to malaria.

The molecular and cellular basis of this association has been revealed in recent studies, showing that malaria-induced hemolysis impairs neutrophil killing of *Salmonella* [41]. Hemolysis results in the liberation of heme, a highly toxic pro-oxidant that is degraded to non-toxic derivatives by heme oxygenase-1 (HO-1). In malaria-infected mice, heme and HO-1 cause release of immature neutrophils into the circulation. These neutrophils are deficient in their production of reactive oxygen species (ROS), which are essential for killing *Salmonella*, allowing the bacteria to proliferate and disseminate inside neutrophils. Significantly, in Gambian children, HO-1 levels are raised and neutrophil function is impaired for many weeks after malaria treatment, creating a potential niche for *Salmonella* outgrowth [42]. Taken together with evidence of the importance of neutrophils in controlling *Salmonella* at the intestinal mucosa and the fact that IL-10 compromises control of *Salmonella* in the gut during malaria co-infection [43,44], these studies indicate a direct causal association between malaria infection, hemolytic anemia, and potentially fatal bacteremia. Thus, even in the absence of high density or symptomatic infection, chronic low-density infections and accompanying hemolysis have potential to cause neutrophil dysfunction, contributing to the high burden of invasive bacterial disease and its associated mortality.

### Malaria Infection Impairs Cognitive Function and School Performance

Another long-term and usually unquantified consequence of malaria infection is the effect on cognitive function, schooling, and social capital development. In addition to the known risk of neurological impairment following cerebral malaria [45], there is increasing evidence linking both uncomplicated febrile episodes and “asymptomatic” malaria infections to impaired cognitive function and reduced educational performance (see below).

Although indirect, uncontrolled, and subject to confounding, consistent evidence that educational outcomes have improved over time in countries that eliminated or substantially controlled malaria—including Sri Lanka, Brazil, Colombia, Ethiopia, Italy, Mali, Mexico, Paraguay, and Uganda [46–51]—is intriguing.

Observational studies provide further evidence that malaria infection leads to cognitive impairment. Most of these studies examined the cognitive detriments associated with symptomatic malaria infection, although one study in Uganda found that “asymptomatic” malaria parasitemia is associated with poor performance in tests of sustained attention and abstract reasoning [52]. A larger body of evidence shows that symptomatic, uncomplicated malaria attacks in school-aged children have been associated with decreased cognitive function, including lower scores in math and language tests in Sri Lanka and Brazil [53–55], lower educational achievement and cognitive performance in Mali [51], and lowered performance in cognitive tests in Yemen [56]. In Uganda and Zambia, episodes of clinical malaria and malaria-related anemia have been associated with reduced cognitive skills by ages three to six years [57–59].

The strongest evidence on the benefits of treatment of both symptomatic and “asymptomatic” malaria, however, comes from randomized, controlled trials of malaria chemoprevention that report on educational outcomes and impact on cognitive performance. In Sri Lanka, a randomized trial showed that nine months of chloroquine prophylaxis administered in schools led to improved educational attainment and decreased absenteeism [60]. These findings are consistent with studies in schoolchildren in Western Kenya and Mali, where Intermittent presumptive treatment was associated with increased sustained attention scores [61,62]. Finally, longer-term benefits to education and cognitive development have been reported from The Gambia, where teenagers who received malaria chemoprophylaxis in early childhood showed higher levels of cognitive function and lower rates of school drop-out, as compared to a control group [63].

### Summary of Evidence

**Fig 1:**
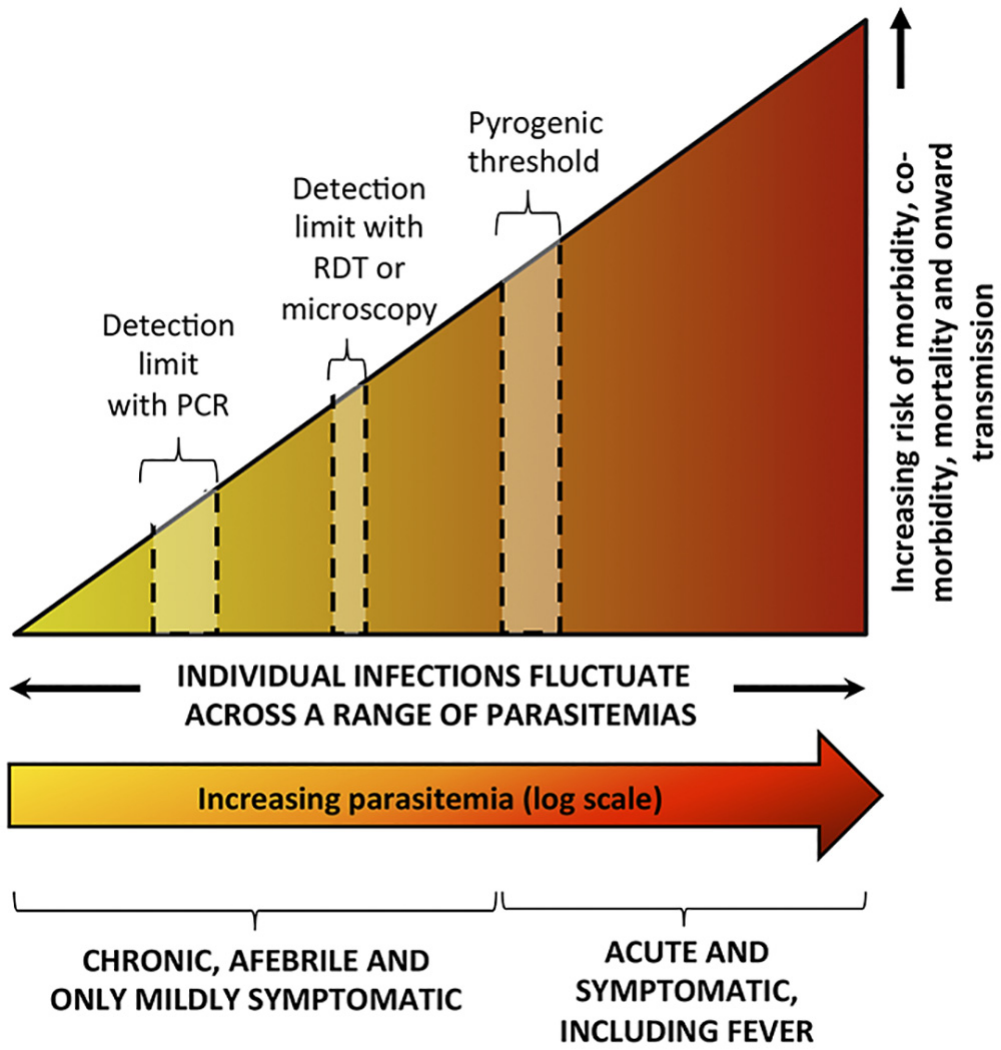
Spectrum of malaria infection. The figure shows increasing risks of morbidity, co-morbidity, mortality, and onward transmission as density of parasitemia increases. Note that malaria RDTs or microscopy are unable to detect low-density chronic infections below the threshold indicated and that infections of very low density are undetectable by PCR [64].

Malaria infections at any density have serious consequences, with rising densities of parasitemia increasing the risks of morbidity, co-morbidity, mortality, and onward transmission (Fig 1). Because low-density infections are not benign, we suggest that the term “asymptomatic malaria infection” is a misnomer and should be characterized as “chronic malaria infection.” A policy change to target all malaria infections with curative treatment offers potential benefit to individuals and society as a whole.

## Challenges and Implications

Treatment of the chronic reservoir of malaria infection carries tremendous programmatic implications. Chasing all malaria infections requires that active, intensive strategies be undertaken, as most chronic infections in malaria-endemic countries currently remain undetected and untreated in the community. The reasons for this are primarily a result of health care–seeking behavior, because in the absence of acute symptoms, most infected people do not present at health facilities. Those who do present to health facilities are still likely to remain undiagnosed, as malaria tests may not be conducted in the absence of febrile symptoms, and diagnostic tests such as RDTs and microscopy only detect infections with sufficient parasite densities (Fig 1).

Active strategies to find and/or treat asymptomatic infections in sub-Saharan African countries currently exist for small, well-defined groups; Intermittent Preventive Treatment in pregnancy for pregnant women, Intermittent Preventive Treatment in infants for children under the age of one year, and seasonal malaria chemoprevention for children under the age of five in areas of highly seasonal malaria are strategies recommended by the WHO [65–67]. A fourth strategy, Intermittent Preventive Treatment in schoolchildren, has a growing body of evidence but is not yet recommended by the WHO [68]. These are all presumptive treatment strategies in which diagnostic testing is not a prerequisite to receiving treatment. In low endemic settings, active case detection is carried out within high-risk communities and in the environs of the homes of febrile malaria patients. People at risk of malaria are screened with a diagnostic test and those testing positive for malaria are treated. In most settings, the diagnostic tests used, namely RDTs and microscopy, will miss 30%–50% of infections that are detectable by PCR [2], which in turn will miss a smaller proportion of very low-density infections (Fig 1). In order to improve the proportion of those infected with malaria that actually receive treatment, studies are underway to evaluate delivery of mass drug administration to high-risk villages [69,70] and, in a more targeted way, treating high-risk households [70,71]. Strategies that only address parasites in humans will reduce the immediate impact of chronic malaria by immediately eliminating the parasite from the human host (assuming the correct drug combinations are used), but unless accompanied by intensive vector control, they are unlikely to have an impact on risk of infection in the future [72]. It is clear that more work is urgently needed to define the appropriate strategies to reduce the pool of chronic malaria infections across the diversity of epidemiological and ecological settings that support malaria transmission globally.

Whichever strategies are chosen to treat chronic malaria infections, these approaches will be operationally intensive, and thus require a rigorous risk-benefit analysis to ensure that the benefits outweigh the risks and costs in each setting of implementation. Risks include the adverse events of the drug chosen and loss of “premunition” to the individual; increased pressure of drug resistance so that therapies for acute, clinical cases become less effective; and substantial programmatic costs [5]. Benefits, on the other hand, include decreased morbidity and mortality from chronic and acute malaria infection, in addition to short-term prophylaxis to individuals and decreased onward malaria transmission throughout the community. In each implementation setting, the risk-benefit assessment will need to consider local epidemiological context, establishing optimal target populations (entire populations or at-risk groups) and intervention timing. The drugs selected to treat chronic malaria infections must be carefully chosen, ideally being well tolerated and highly effective. Selection for drug resistance should be monitored.

In areas where the benefits of finding, treating, and chasing all malaria infections outweigh the risks, two additional measures must be carried out to ensure the success of intensive efforts. First, strong and appropriate communication strategies should be implemented to engage communities and health care professionals [73]. Secondly, to ensure the prevention of future malaria infection, approaches must be instigated with concomitant vector control measures.

## Conclusion

We believe that malaria infections can no longer be considered as either “symptomatic” or “asymptomatic” based on symptoms such as fever and chills alone. Malaria infections at any density have serious health and societal consequences, with rising parasite densities increasing the risks of anemia, maternal and neonatal mortality, bacterial co-infection, and cognitive impairment.

This is a call for the malaria community to develop strategies for comprehensively tackling the reservoir of chronic human malaria infection. Operational challenges to detecting and treating chronic malaria infections have significant scientific, programmatic, financial, and political implications but promise enormous benefits to public health.

## Abbreviations

HMS: hyper-reactive malarial splenomegaly
HO-1: heme oxygenase-1
ITN: insecticide treated bed-net
NTS: non-typhoid salmonellae
RDT: rapid diagnostic test
ROS: reactive oxygen species
